# Nonhost resistance to rust pathogens – a continuation of continua

**DOI:** 10.3389/fpls.2014.00664

**Published:** 2014-12-11

**Authors:** Jan Bettgenhaeuser, Brian Gilbert, Michael Ayliffe, Matthew J. Moscou

**Affiliations:** ^1^The Sainsbury Laboratory, Norwich Research ParkNorwich, UK; ^2^Commonwealth Scientific and Industrial Research Organisation, Agriculture FlagshipCanberra, ACT, Australia

**Keywords:** host range, disease resistance, nonadapted pathogen, NHR, *formae speciales*, Pucciniales

## Abstract

The rust fungi (order: Pucciniales) are a group of widely distributed fungal plant pathogens, which can infect representatives of all vascular plant groups. Rust diseases significantly impact several crop species and considerable research focuses on understanding the basis of host specificity and nonhost resistance. Like many pathogens, rust fungi vary considerably in the number of hosts they can infect, such as wheat leaf rust (*Puccinia triticina*), which can only infect species in the genera *Triticum* and *Aegilops*, whereas Asian soybean rust (*Phakopsora pachyrhizi*) is known to infect over 95 species from over 42 genera. A greater understanding of the genetic basis determining host range has the potential to identify sources of durable resistance for agronomically important crops. Delimiting the boundary between host and nonhost has been complicated by the quantitative nature of phenotypes in the transition between these two states. Plant–pathogen interactions in this intermediate state are characterized either by (1) the majority of accessions of a species being resistant to the rust or (2) the rust only being able to partially complete key components of its life cycle. This leads to a continuum of disease phenotypes in the interaction with different plant species, observed as a range from compatibility (host) to complete immunity within a species (nonhost). In this review we will highlight how the quantitative nature of disease resistance in these intermediate interactions is caused by a continuum of defense barriers, which a pathogen needs to overcome for successfully establishing itself in the host. To illustrate continua as this underlying principle, we will discuss the advances that have been made in studying nonhost resistance towards rust pathogens, particularly cereal rust pathogens.

## INTRODUCTION

A considerable body of research now exists at the genetic and molecular level on some of the mechanisms underlying nonhost resistance (NHR). This review will focus on what is known regarding the interaction of rust pathogens with their hosts and nonhosts and include key concepts in NHR derived from other plant–pathogen systems. Previous definitions have attempted to qualitatively distinguish between host and nonhost interactions, however, many plant–pathogen systems cannot be neatly classified into these two extremes. In reality a continuum of resistance outcomes is possible ranging from immunity to partial resistance with varying degrees of efficacy. As will be discussed, a variety of different nonhost outcomes occur for rust pathogens and thus, a definition of nonhost is proposed that incorporates the inherent quantitative nature of nonhost status.

## THE RUSTS

Rusts (order: Pucciniales) are an order of obligate biotrophic fungal plant pathogens, of which many are agronomically important and affect major cereal crops such as wheat, barley, rye, and oat, as well as many other economically important plants ranging from legumes like soybean to trees like coffee (Agrios, 2005). The approximately 5,000 species of rust fungi tend to be specialized pathogens of specific host genera, but their life cycles can be very complex (Agrios, 2005). These life cycles range from the simple to the extreme, with the latter exemplified by macrocyclic, heteroecious rust fungi which have life cycles involving up to five different spore stages and two different hosts: a primary host allowing clonal reproduction and an alternate host to complete sexual reproduction (Agrios, 2005).

*Puccinia graminis*, causal agent of stem rust on wheat, barley, and oat, has a complex heteroecious life cycle with species in the Triticeae being telial/uredinial stage primary hosts (Leonard and Szabo, 2005). Asexual reproduction of the dikaryotic urediniospores can lead to epidemics on the primary hosts, with multiple infection cycles occurring during the growing season (Leonard and Szabo, 2005). Under appropriate developmental and unfavorable environmental conditions, the pathogen produces an alternative developmental morphology, telia, which erupt through the host tissue and produce diploid teliospores, which are considered survival structures. Haploid basidiospores are produced by the teliospore and infect an alternate spermagonial/aecial host, which in the case of *Puccinia graminis* are dicot barberry plants (*Berberis* spp.; Roelfs et al., 1992). Infection of barberry with basidiospores leads to the development of the spermagonial stage on the upper side of leaves. Haploid spermatia are formed and in the event that corresponding mating types are present, hyphae penetrate to form dikaryotic aecia on the lower side of leaves (Agrios, 2005). Aeciospores can infect the primary host to produce uredinia and thereby complete the sexual life cycle of the rust. Similar heteroecious life cycles occur for *Puccinia striiformis* and *Puccinia triticina*, the causal agents of stripe rust and wheat leaf rust disease, respectively (Bolton et al., 2008; Hovmøller et al., 2011).

Not all rust fungi share this five-stage (macrocyclic) life cycle, as some species only produce teliospores and basidiospores, i.e., they have a microcyclic life cycle (Agrios, 2005). In addition, not all macrocyclic rusts are heteroecious (i.e., need two different hosts to complete their life cycle). Autoecious rusts, such as the asparagus rust pathogen *(Puccinia asparagi)* and flax rust pathogen (*Melampsora lini*), complete their life cycle on a single host (Agrios, 2005).

The evolution of a heteroecious life cycle by some rusts, such as *Puccinia graminis*, is of interest, as it has clearly required a species jump by the progenitors of these modern pathogens. It is believed that the progenitors of cereal rusts parasitized dicot plants of the ancestral Berberidaceae in what is now northern Europe prior to the evolution of the *Mahonia* and *Berberis* genera in this family (Leppik, 1961; Wahl et al., 1984). The grasses subsequently evolved in the tropics and radiated out, speciating during this process. Eventually contact between rust infected *Berberis* plants and grasses occurred, enabling the evolution of modern heteroecious rust species that parasitize the Poaceae. One can only speculate if the initial parasitism of the grasses in this scenario required the pathogen to overcome an active NHR response or if these plants, which had never previously encountered a rust pathogen, were incapable of recognizing this pathogen. Rice being a tropical grass and a nonhost of all rust pathogens is unlikely to have been exposed to rust pathogens until relatively late, however, as described below, this plant species mounts an active defense response upon challenge with cereal rusts (Ayliffe et al., 2011). These data suggest that extended plant–rust coevolution is not a prerequisite for NHR recognition of rust pathogens.

## *Formae speciales* – EVOLUTION IN ACTION

Many cereal rust species consist of subgroups, or *formae speciales* (ff. spp.), that have specialized to infect only certain plant species amongst the entire range of host plant species parasitized by the pathogen. The formae speciales concept was first introduced by Eriksson (1894). After inoculating a range of grass species with rust isolates obtained from different host plants, he was able to determine limitations on the host range of different rust isolates within the species. In the case of *Puccinia striiformis*, he identified five ff. spp., or specialized forms of rust in this species, these being *Puccinia striiformis* f. sp. *tritici*, *Puccinia striiformis* f. sp. *hordei*, *Puccinia striiformis* f. sp. *secalis*, *Puccinia striiformis* f. sp. *elymi* and *Puccinia striiformis* f. sp. *agropyri*, with each *forma specialis* largely restricted to infecting only plant hosts of the Triticeae, *Hordeum* spp., *Secalis* spp., *Elymus* spp., or *Agropyron* spp., respectively (Eriksson, 1894). In addition, Eriksson (1894) demonstrated that similar ff. spp. existed within the stem rust pathogen *Puccinia graminis*. Rust pathogen ff. spp. presumably represent the early stages of the evolution of new rust species and demarcation of the respective host and nonhost plant species of these rusts.

## RUST HOST RANGES

The host range of a pathogen is defined by the plant species it can infect and successfully complete its life cycle on (Thordal-Christensen, 2003; Schulze-Lefert and Panstruga, 2011). Some rust species such as *Puccinia hordei* (barley leaf rust), *Puccinia sorghi* (maize rust) and *Puccinia kuehnii* (sugarcane rust) have restricted host ranges confined to a few species in one or two genera (Wahl et al., 1984). A similar limited host range is apparent for *Puccinia triticina*, which infects hexaploid wheat and other wheat species from the genus *Triticum* (Roelfs et al., 1992), as well as several *Aegilops* species (Yehuda et al., 2004; Bolton et al., 2008), as its primary telial/uredinial hosts.

In contrast, the host range of *Puccinia graminis* is large and includes 365 species of plants in 54 genera, while that of *Puccinia coronata* (oat crown rust) includes 290 host species belonging to 72 genera (Wahl et al., 1984). Similarly, the natural host range of *Phakopsora pachyrhizi* (Asian soybean rust) is large and the pathogen can complete its life cycle on 31 species from 17 leguminous plant genera (Ono et al., 1992). Additional artificial inoculation studies confirmed that this rust species completes its life cycle on another 60 species from 26 genera, largely within the Papilionoideae, although care must be exercised when generalizing from artificial versus natural inoculations. More recent studies added a further 65 species from 25 genera, including 12 previously unreported genera, to this list of plants that allow infection or life cycle completion upon artificial inoculation with *Phakopsora pachyrhizi* (Slaminko et al., 2008). Further research will show whether those species that allow some degree of infection form part of the host range of *Phakopsora pachyrhizi*.

This large diversity in host range size is of significant interest. Why have some rust species evolved the ability to parasitize so many different hosts? Is this due to a series of aggressive host jumps or the development of a virulence arsenal more generically adapted for plant colonization? Conversely, is there extensive variation in plant species for resistance genes to nonhost rust pathogens? Alternatively, it may be the rapid evolution of a progenitor host species and the coevolution of the rust. Do rust species with a single host reflect the last remnants of a plant–pathogen interaction with both the plant and specialized pathogen headed for eventual extinction?

In the case of rust species the definition of host range is complicated by the elaborate life cycle of the pathogens. Host range in heteroecious rust species like cereal rusts are further expanded by an alternate host. As described above, the dicot hosts of cereal rusts are thought to be the original hosts. While many alternate hosts are known, our knowledge is still limited. Despite decades of searching for the alternate host of *Puccinia striiformis*, it was only recently that barberry was identified (Jin et al., 2010). A similar situation exists with *Puccinia triticina*, where the pathogen has been found to use different species of the genera *Thalictrum* and *Isopyrum* as alternate hosts (Roelfs et al., 1992; Bolton et al., 2008). These interactions between *Puccinia triticina* and the alternate hosts seem to be geographically restricted, complicating the understanding of host range in this context (Bolton et al., 2008). Regional adaptation to different alternate hosts was also reported for *Puccinia graminis* (Jin et al., 2014). North American *Puccinia graminis* f. sp. *tritici* populations east of the Rocky Mountains commonly use *Berberis vulgaris* as alternate host (Roelfs et al., 1992; Jin et al., 2014), yet it is only rarely found on this host in the northwestern United States (Jin et al., 2014). By isolating *Puccinia graminis* from aecia on *Mahonia repens* and *Mahonia aquifolium* and inoculating various wheat lines and *Elymus glaucus*, Jin et al. (2014) demonstrated how *Puccinia graminis* can maintain its virulence diversity in this region and also bypass agricultural selection pressure by completing its life cycle on *Mahonia* and *Elymus*. These two examples demonstrate our increasing understanding of the complete host range of these pathogens, especially concerning their potential host ranges on alternate hosts.

## SPECIES JUMPS – NEW HOSTS

In a study of 80 Pucciniaceae taxa, which collectively parasitize hosts in 33 angiosperm families, evidence for both coevolution of host and pathogen was observed in addition to numerous possible examples of host species jumps involving both telial and aecial forms of the pathogen (van der Merwe et al., 2008). These host jumps occurred on taxonomically unrelated plant species that were geographically associated with the pathogen (van der Merwe et al., 2008).

Similarly, de Vienne et al. (2013) conclude that cospeciation of pathogens and their hosts occurs over a short evolutionary time period, whereas the long-term evolution of plants and pathogens involves frequent host species jumps. From an extensive analysis of published literature they conclude that only 7% of cases represented convincing examples of cospeciation and host shifts constituted the most frequent form of pathogen speciation (de Vienne et al., 2013). This review included one rust study on three flower-mimic rusts (*Puccinia monoica, Puccinia thlaspeos,* and *Puccinia consimilis*) and their host genera (Roy, 2001). Flower-mimic rusts sexually reproduce by inducing the formation of pseudoflowers in their hosts, which facilitate fertilization of the rusts by attracted insects (Roy, 1993). Potentially influenced by localized rainfall differences, short distance dispersal of sexual spores by insects and proximity of potential hosts, geographic distance rather than phylogenetic distance predicted host jumps in this system (Roy, 2001).

However, very recent examples of changes in rust host range do exist, which are likely to be a direct consequence of agricultural practices. The cultivation of Australian eucalyptus species in South America has enabled these plants to be parasitized by a rust species endemic to this continent, *Puccinia psiidi*, which was not present in Australia. This rust has now unfortunately arrived in Australia with many of the native plants susceptible to this pathogen, with growth occurring on 107 species in 30 genera (Carnegie and Lidbetter, 2012). Apart from Australian flora, this pathogen attacks more than 129 species in 33 genera of the Myrtaceae (Carnegie and Lidbetter, 2012). The parasitism of eucalyptus species by this rust probably does not constitute a host jump in the true sense, but rather a host expansion via an opportunistic introduction to susceptible species not previously exposed to this pathogen.

In some rare cases somatic hybridization of two rusts species or ff. spp. has produced a hybrid pathogen with an expanded host range (Park and Wellings, 2012). For example, a *Puccinia graminis* hybrid lineage was formed by hybridization of *Puccinia graminis* f. sp. *tritici* and *Puccinia graminis* f. sp. *secalis* to produce a hybrid rust with new virulence specificities (Burdon et al., 1981, 1982). Similarly, a hybrid rust between *Melampsora medusae* and *Melampsora larici-populina* was reported in New Zealand that had a virulence spectrum distinct to that of either presumptive parent (Spiers and Hopcroft, 1994).

Sexual recombination events have not been conclusively recorded between rust taxa apart from two potential cases. In one case a potential hybrid between *Melampsora medusae* and *Melampsora occidentalis*, called *Melampsora x columbiana*, was suggested to have arisen by this process, although somatic hybridization followed by some degree of parasexuality could not be excluded (Newcombe et al., 2000, 2001). Similarly, some limited evidence exists for sexual recombination occurring between North American pine blister rusts, specifically between *Cronartium comandrae,* an endemic species, and *Cronartium ribicola,* an exotic species (Joly et al., 2006).

Interestingly, the parasitism of new hosts is not always dependent upon major changes in pathogen biology such as somatic hybridization. For example, the specialization of *Phytophthora* into the species *Phytophthora infestans* and *Phytophthora mirabilis*, infecting *Solanum* spp. and *Mirabilis jalapa*, respectively, is directly reflected in a mutation of a single fungal gene (an effector gene – see below) that is associated with a host jump 1,300 years ago (Dong et al., 2014).

## RUST – HOST INTERACTIONS: LEVELS OF RESISTANCE

**FIGURE 1:**
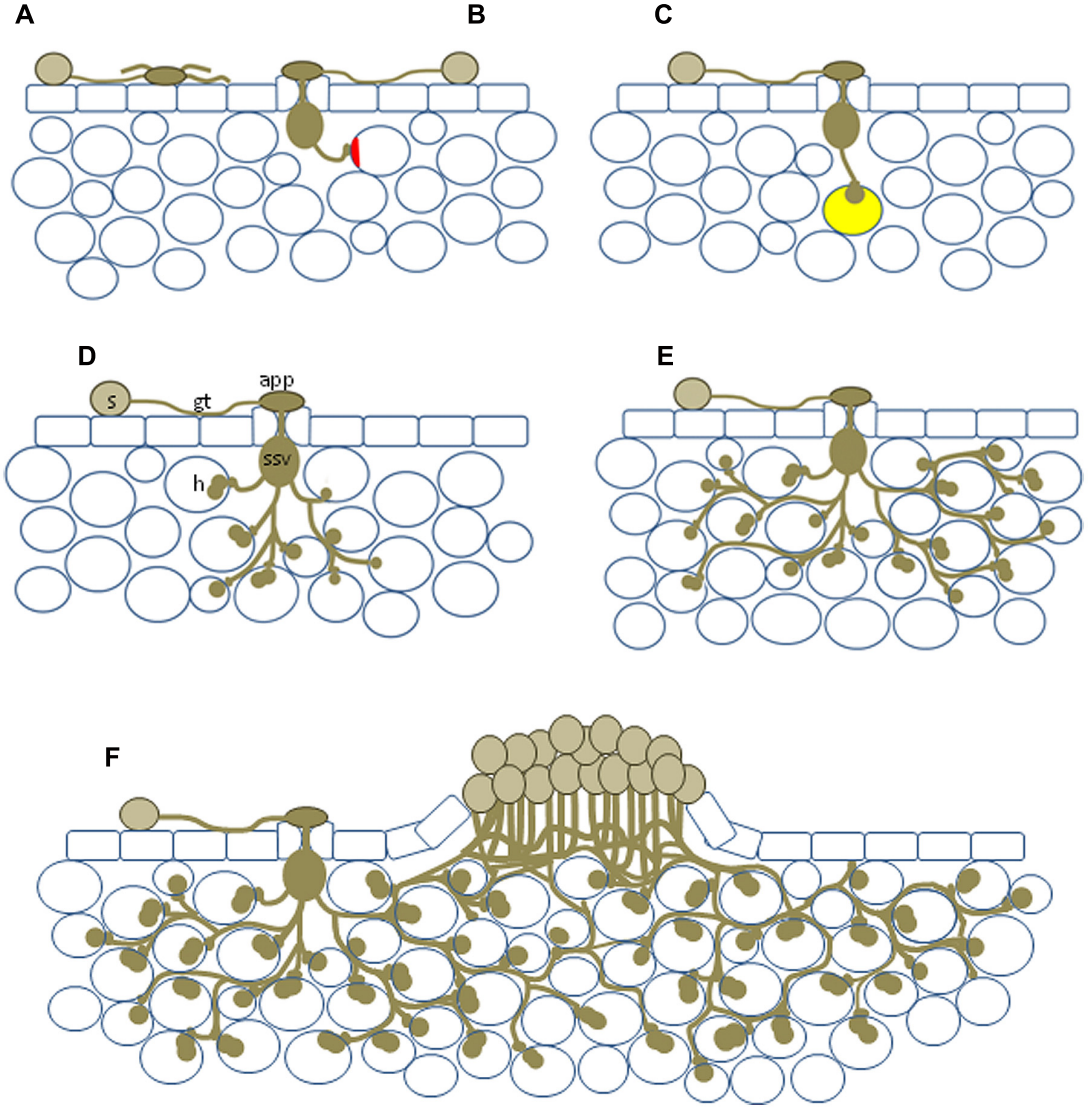
**Diagram showing the range of potential NHR outcomes. (A)** Basic incompatibility in which a spore germinates to produce an aberrant appressorium-like structure in the absence of a stoma; **(B)** pre-haustorial resistance in which a germination event enters the leaf but is unable to penetrate the cell wall, plant cell wall appositions (shown in red) can occur; **(C)** infection resulting in the formation of a single haustorium, autofluorescence (yellow) can be associated with these events; **(D)** haustoria produced in multiple plant cells; **(E)** relatively large infection site encompassing numerous mesophyll cells but sporulation never observed; **(F)** formation of a sporulating uredinia, usually much smaller than those observed on susceptible host plants. s, urediniospore; gt, germ tube; app, appressorium; ssv, substomatal vesicle; h, haustorium.

**FIGURE 2:**
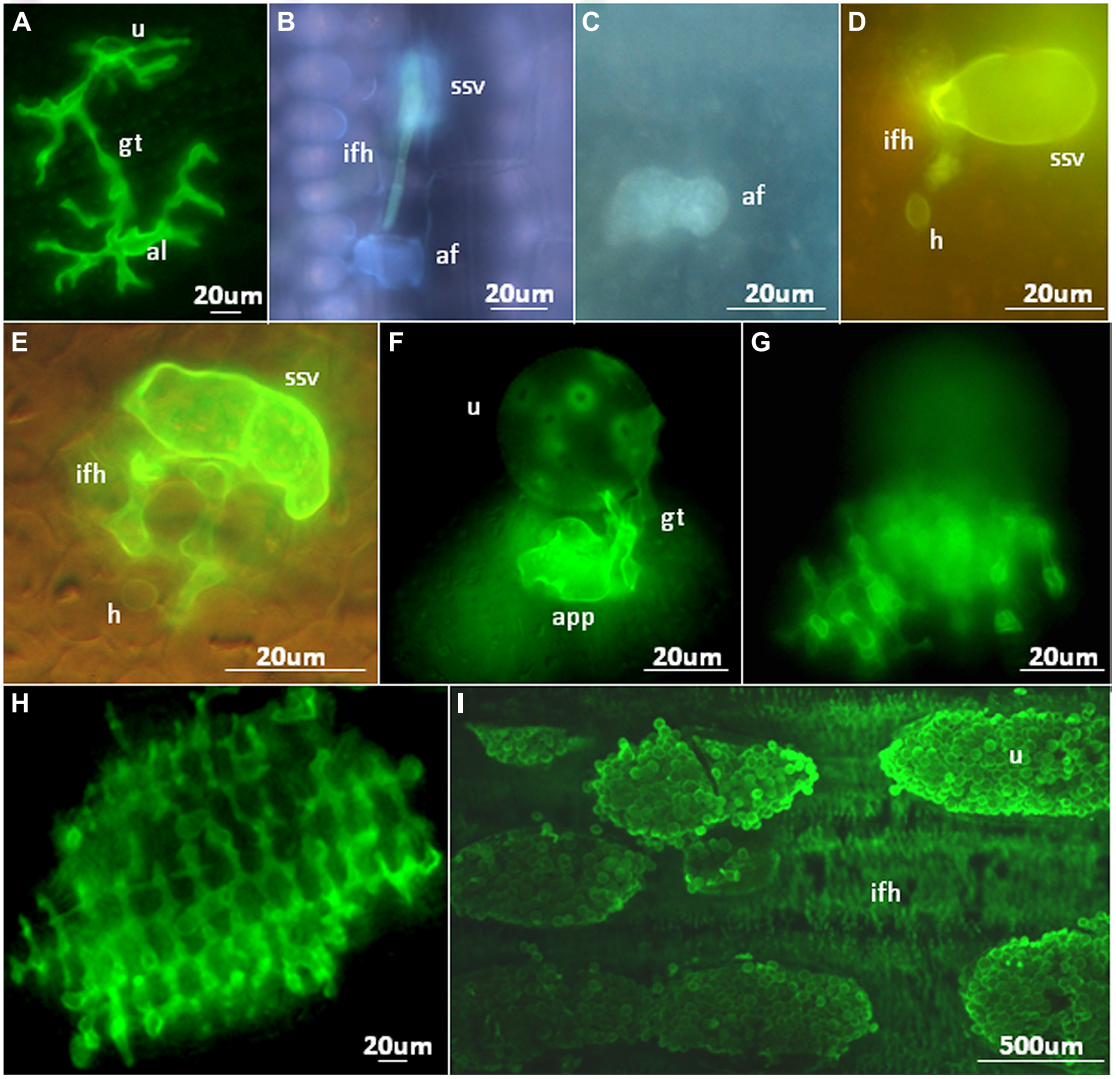
**Microscopic analyses of NHR outcomes to rust pathogens. (A)** Growth of a *Melampsora lini* (flax rust) germ tube on the surface of a rice leaf. An aberrant appressorium-like structure (al) has been produced. **(B)** Pre-haustorial resistance against a *Puccinia graminis* f. sp. *tritici* infection site on *Setaria italica*. Contact of a fungal infection hyphae with a single mesophyll cell results in autofluorescence. **(C,D)** An autofluorescent *Brachypodium distachyon* mesophyll cell **(C)** containing a *Puccinia striiformis* f. sp. *tritici* haustorium **(D)**. **(E)** A *Puccinia hordei* infection site on rice with a single, non-autofluorescent mesophyll cell containing a haustorium. **(F,G)** A *Puccinia striiformis* f. sp. *tritici* urediniospore on the surface of a rice leaf that has produced an appressorium **(F)** and underlying infection hyphae **(G)** that encompass multiple mesophyll cells. **(H)** A *Puccinia graminis* f. sp. *tritici* infection site on a rice leaf producing infection hyphae that encompass numerous mesophyll cells. Each dark, circular structure surrounded by green stained fungal infection hyphae is a single mesophyll cell. **(I)** Multiple *Puccinia striiformis* f. sp. *tritici* uredinia on a *Brachypodium distachyon* leaf producing urediniospores with underlying infection hyphae also apparent. af, autofluorescent plant cell; gt, spore germ tube; ifh, infection hyphae; h, haustoria; ssv, substomatal vesicle; u, urediniospore. All microscopic images were produced as described by Ayliffe et al. (2011).

A rust that is capable of parasitizing a plant species is said to be an adapted pathogen of that species, i.e., it can form all the necessary cellular components for colonization and successful reproduction. This same rust species will be incapable of parasitizing the vast majority of plant species for which it is a nonadapted pathogen. Infection of a host plant by urediniospores from an adapted rust pathogen in many cases involves germination of the spore on the leaf surface and growth of a germ tube across the leaf surface, whereupon it identifies a plant stoma by a thigmotropic response, leading to the production of an appressorium over the stoma (**Figures 1D** and **2F**). (Note: although **Figures 1** and **2** depict NHR outcomes the same fungal structures are produced during host infection.) From the appressorium an infection peg is inserted between the stomatal guard cells and a substomatal vesicle is produced within the leaf apoplast. Some rust species (e.g., *Phakopsora pachyrizi*) enter the host plant by germinated urediniospores forming an appressorium on the leaf surface and directly penetrating through the plant epidermis with an appressorium, and subsequently the hypha infect intercellular space. Infection of the alternate hosts of cereal rusts is also performed in this latter manner.

After substomatal vesicle formation, infection hyphae emerge from this vesicle and ramify through the apoplastic space, inserting haustoria into adjacent plant cells as they extend outward (**Figures 1D** and **2G,H**). Haustoria are specialized fungal feeding structures that arise from haustorium mother cells and penetrate the plant cell wall and invaginate, but do not penetrate, the plant cell membrane (**Figures 2D,E**). Nutrient transport and molecular trafficking occurs across this plant–pathogen interface called the extrahaustorial matrix (Staples, 2001; Kemen et al., 2005; Garnica et al., 2014). The infection site established from the urediniospore continues to expand and colonize additional plant cells. New urediniospores develop from fungal pedicels, called uredinia, which emerge from the center of the infection site. The uredinia erupt through the leaf surface and thousands of urediniospores are wind dispersed to repeat this asexual infection cycle (**Figures 1F** and **2I**). Given the relative simplicity of the asexual cycle compared with the sexual cycle and that asexual colonization is the causal route of disease on cereals this stage is the most extensively characterized plant–rust interaction.

Successful infection of a plant host by rust requires the pathogen to overcome numerous defense barriers. The first barrier, while not a plant defense mechanism *per se*, is nonetheless a real obstacle to nonadapted rust pathogen infection and that is a requirement of basic compatibility. A consequence of host–pathogen coevolution is that pathogens can specialize to such a degree they have difficulty in effectively recognizing other plant species as potential hosts. In this case the majority of germinated spores are incapable of identifying a stoma on nonhost plant leaves, a key requirement for appressorium production (see below). It is noteworthy that numerous rust pathogens can be induced to produce appressoria on simple membranes with suitable sized ridges, suggesting leaf topography is a major determinant in this process (Dickinson, 1949; Heath, 1977; Hoch et al., 1987; Allen et al., 1991). For those rusts like *Phakopsora pachyrizi* that enter the leaf by directly penetrating the plant epidermis hydrophobicity or wax signals play a role in inducing pre-penetration structures, rather than thigmotropism (Uppalapati et al., 2012; Ishiga et al., 2013).

The plant epidermis constitutes a formidable defense to potential pathogens. In the case of mildew pathogens, which also directly penetrate the epidermis, it is a significant barrier to nonadapted mildews. Three genes in *Arabidopsis thaliana*, *PEN1, PEN2,* and *PEN3 (PENETRATION)*, which encode a syntaxin vesicle targeting protein, glucosyltransferase, and ATP-binding cassette (ABC) transporter, respectively, are essential components of this epidermal cell-based defense mechanism, which when perturbed enables nonadapted mildew pathogen species to penetrate the leaf (Collins et al., 2003; Lipka et al., 2005, 2010; Stein et al., 2006; Underwood and Somerville, 2013). Underlying penetration resistance is a second layer of defense mediated by components of salicylic acid (SA) based responses. *Arabidopsis thaliana* plants with mutations in both penetration resistance genes and genes in this second layer of defense can become hosts for nonadapted mildew pathogens such as the pea mildew pathogen *Erysiphe pisi* (Stein et al., 2006). As will be discussed in depth below, these *PEN* genes play a role in penetration resistance to the rust pathogen *Phakopsora pachyrhizi* on nonhost plants, confirming common epidermal defense mechanisms against these diverse pathogen species.

In some pathosystems preformed chemicals play a role in defining host range with nonadapted pathogens unable to overcome these chemical defenses. For example, oats produce antimicrobial triterpene glycosides (avenacins) which effectively make it a nonhost to *Gaeumannomyces graminis* var. *tritici,* the causal agent of take-all disease in wheat and barley (Bowyer et al., 1995; Papadopoulou et al., 1999; Qi et al., 2004). As yet preformed chemical barriers have not been demonstrated as a major constraint on rust parasitism.

Active plant defense mechanisms must also be overcome for successful rust parasitism to occur. Extensive research on host resistance in numerous plant pathosystems has given rise to a two-layered paradigm of the active plant defense systems, consisting of pathogen-associated molecular pattern (PAMP) or damage associated molecular pattern (DAMP) triggered immunity (PTI/DTI) and effector-triggered immunity (ETI; Jones and Dangl, 2006). PAMPs are highly conserved, indispensible pathogen molecules which include chitin and xylanases in the case of fungal pathogens (Zipfel, 2008), while DAMPs are endogenous plant molecules released during interactions with pathogens (Boller and Felix, 2009). Recognition of PAMPs or DAMPs via membrane localized pattern recognition receptors (PRRs) leads to a signaling cascade that alerts the plant to the presence of a pathogen and induces PTI (Ishiga et al., 2013). This PTI response frequently involves ion fluxes, the production of reactive oxygen species, protein phosphorylation, ethylene biosynthesis, and callose deposition (Boller and Felix, 2009).

The successful colonization of a plant host requires that the rust pathogen must suppress PTI which is achieved, as for many other pathogens, by the deployment of hundreds of small, secreted effector proteins into plant tissues (Giraldo and Valent, 2013). Rust pathogen effector proteins are produced by haustoria and secreted into the extrahaustorial matrix (i.e., the space between the haustorium cell wall and the plant cytoplasmic membrane) from where many are thought to move into the plant cytoplasm. These effectors likely suppress PTI and alter plant cell homeostasis for the pathogens’ benefit, as observed for other pathogenic fungi (Rafiqi et al., 2010; Giraldo and Valent, 2013). Predicted effector complements of rust pathogens are diverse and presumably play a large role in defining their host range. Amongst different rust species common predicted effector proteins can be identified, although their sequence divergence is high, with more related rust pathogens having more similar effector complements, although each rust species also contains a complement of unique effector proteins (Duplessis et al., 2011; Nemri et al., 2014).

However, not all plants within a given species are susceptible to all isolates of a rust pathogen. As for other pathosystems, plants have evolved resistance proteins, typified by nucleotide binding site–leucine rich repeat (NBS–LRR) proteins, that each recognize a specific rust effector, called an avirulence (Avr) protein. Upon recognition, a more extreme defense response termed ETI is activated, which frequently involves hypersensitive cell death. NBS–LRR proteins that recognize adapted rust pathogens have been isolated from flax (Lawrence et al., 1995, 2010; Anderson et al., 1997; Dodds et al., 2001a,b), maize (Collins et al., 1999; Webb et al., 2002), wheat (Feuillet et al., 2003; Huang et al., 2003; Cloutier et al., 2007; Loutre et al., 2009; Periyannan et al., 2013; Saintenac et al., 2013), and barley (Wang et al., 2013). The best characterized interactions between rust effectors and plant NBS–LRR resistance proteins are from the flax rust pathogen, *Melampsora lini*, and the flax plant, *Linum usitatissimum,* where some NBS–LRR proteins recognize flax rust effector proteins by direct protein–protein interaction (Dodds et al., 2006; Catanzariti et al., 2010). In most pathosystems effector recognition by R (resistance) proteins is indirect and involves detection of specific modifications of host proteins mediated by the effector (i.e., guard hypothesis). As individual effectors are often dispensable, different isolates of a rust pathogen species have different effector complements, generally consisting of allelic variants rather than novel genes. In addition, different members of a host plant species contain different *R* gene complements. Rust infections can therefore lead to either a resistant or a susceptible outcome, depending upon the plant and pathogen genotypes involved. These molecular interactions form the basis of the gene-for-gene hypothesis first formulated by Flor (1942) using the flax-flax rust system.

In addition to NBS–LRR encoding rust resistance genes, several adult plant resistance (APR) genes have been cloned which were originally identified as quantitative trait loci (QTLs) with moderate rust resistance effects at the adult plant stage. One example is the *Lr34/Yr18/Sr57/Pm38* gene of wheat which confers resistance to a range of pathogens, including *Puccinia triticina*, *Puccinia striiformis* f. sp. *tritici, Puccinia graminis* f. sp. *tritici*, and *Blumeria graminis* f. sp. *tritici* (wheat powdery mildew; Dyck et al., 1966; Krattinger et al., 2009; Spielmeyer et al., 2013). *Lr34* is remarkable in that it provides resistance to all tested isolates of each pathogen and has not been overcome during decades of deployment. This APR gene encodes an ABC transporter protein, although the substrate of this protein is unknown (Krattinger et al., 2009). A second APR gene to be cloned is the *Yr36* gene that confers broad spectrum resistance to *Puccinia striiformis* f. sp. *tritici* (Fu et al., 2009). This gene encodes a protein with an N-terminal kinase domain fused to a C-terminal steroidogenic acute regulatory protein-related lipid transfer domain (START domain). The mechanism of resistance mediated by *Yr36* is largely unknown. In some cases combining of APR genes can lead to increased resistance, i.e., these genes have additive effects, which appears to be the case for the *Lr34* and *Yr36* genes (Uauy et al., 2005).

## NONHOST RESISTANCE TO RUST PATHOGENS

Despite the omnipresence of potential pathogens in the environment and the constant threat of infection, disease is the exception, not the rule, and generally the vast majority of plants are healthy. This is due to the fact that plants can only be infected by a very limited number of the potential pathogens present in the environment. It therefore becomes apparent that all plants are nonhosts to the vast majority of pathogens, which highlights the effectiveness of NHR in the vast majority of cases (Heath, 1991).

Ideally a species would be classified as a nonhost, if all accessions of the species were resistant to all isolates of the pathogen and uniform levels of resistance were observed in all interactions between both species. In practice, delimiting the host range of a pathogen is complicated by the quantitative nature of phenotypes in the transition from host to nonhost. Interactions in this transitional phase may involve only a few accessions of a species being infected by a pathogen or only some isolates of a pathogen being able to infect a plant species. In addition, a continuum of disease phenotypes exists in the interaction with different plant species, which range from full susceptibility (host) to complete immunity (nonhost). The following examples demonstrate some of the phenotypic outcomes that can occur between nonadapted rust pathogens and nonhost plant species.

### *Arabidopsis thaliana* AND NONADAPTED RUST PATHOGENS

Several groups have focused their attention on the interaction of the model plant *Arabidopsis thaliana* with several nonhost pathogens. Germination of *Puccinia triticina* urediniospores on *Arabidopsis thaliana* was similar to that on wheat (91% on *Arabidopsis thaliana* compared to 95% and 93% on susceptible and resistant wheat varieties, respectively), but identification of stomata and appressoria formation were significantly reduced (12% on *Arabidopsis thaliana* compared to 85% and 83% on susceptible and resistant wheat varieties, respectively; Shafiei et al., 2007). As 62% of pathogen penetration attempts on *Arabidopsis thaliana* lead to guard cell death that prevented further fungal growth, only 0.2% of the urediniospores managed to form haustoria within leaf mesophyll cells (compared to 72% and 4% on susceptible and resistant wheat varieties, respectively). These numbers did not increase when testing *Arabidopsis thaliana* mutants defective in defense-related pathways. Using natural variation between *Arabidopsis thaliana* accessions Col-0 and L*er*, Shafiei et al. (2007) identified three QTLs that controlled 41% of sub-stomatal vesicle frequency and two QTLs controlled 21% of guard cell death. Several of these loci co-segregated with genes encoding NBS–LRR or receptor-like kinase (RLK) proteins (Shafiei et al., 2007).

Complementary to the results with *Puccinia triticina*, stomatal penetration frequencies of *Arabidopsis thaliana* by three *Puccinia striiformis* f. sp. *tritici* isolates was also found to be significantly lower than those observed for wheat, however, no haustoria were formed following penetration (Cheng et al., 2013). The authors attribute this to an active defense response involving callose deposition and accumulation of antimicrobial phenolic compounds, but found no signs of reactive oxygen species release. However, unlike the *Puccinia triticina* study (Shafiei et al., 2007), increased hyphal growth of the stripe rust pathogen lead to the occasional formation of haustoria in *npr1-1 (non-expressor of PR genes)* mutant *Arabidopsis thaliana* plants and in plants depleted in SA, an important plant hormone involved in defense responses (Cheng et al., 2013). Additionally, transcription of the SA-response genes *PR1b* and *PR5* and the jasmonic acid (JA) response gene *PDF1.2* was up-regulated during infection of wild type Col-0, although at different time points during infection.

Nonhost resistance of *Arabidopsis thaliana* has also been examined with other rusts, such as *Phakopsora pachyrhizi* (described below), *Hemileia vastatrix,* causal agent of coffee leaf rust, and *Uromyces vignae*, causal agent of cowpea rust (Mellersh and Heath, 2003; Azinheira et al., 2010). *Hemileia vastatrix* penetrated *Arabidopsis thaliana* via stomata, but was unable to form haustoria for nutrient uptake (Azinheira et al., 2010). Similar to the other nonadapted rust infections of *Arabidopsis thaliana*, penetration of Col-0 by *Hemileia vastatrix* also lead to a hypersensitive response of the guard cells, callose deposition in epidermal and mesophyll cells, as well as a build-up of phenolic compounds. Moreover, expression of *PR1b* peaked at 18 h post-inoculation, whereas expression of *PDF1.2* peaked at 42 h post-inoculation (Azinheira et al., 2010). Mellersh and Heath (2003) infected 17 *Arabidopsis thaliana* accessions with *Uromyces vignae* and all but one accession displayed pre-haustorial NHR. Haustoria formation, however, increased in mutants defective in the SA pathway, which lead to callose deposition around haustoria. The authors conclude that initial defense gene expression limits fungal growth, with an additional SA-dependent layer preventing haustoria formation (Mellersh and Heath, 2003).

### WHEAT RUST ON BEANS AND BEAN RUST ON WHEAT

Similar observations to those observed in *Arabidopsis thaliana* upon cereal rust infection were made in broad bean (*Vicia faba*) upon infection with *Puccinia striiformis* (Cheng et al., 2012). Only 1.2% of germinated urediniospores were able to locate a stoma on the leaf surface. However, having located a stoma, the majority (96%) of these infection attempts then entered the leaf. Many of these infection sites within the leaf showed aberrant substomatal vesicle morphology and only 2% of infection hyphae produced haustoria. Infection sites were associated with hydrogen peroxide production, callose deposition, and upregulation of SA responsive genes like *PR1*. Plant cells containing haustoria were autofluorescent, consistent with a hypersensitive cell death response (Cheng et al., 2012).

A similar response was observed when the bean rust pathogen, *Uromyces fabae*, was inoculated on wheat (Zhang et al., 2011). Again, germ tubes from very few (2%) germinated spores were able to identify a stoma and produce an appressorium. Of the few infection sites that successfully entered the leaf, only 4% managed to produce haustoria and these infected mesophyll cells became callose encased, although cell death was not observed. Most infection hyphae that entered the leaf apoplast were blocked when contact was made with mesophyll cells and cell wall appositions were formed. Reactive oxygen species were present at infection sites.

Limited haustoria formation was also observed on nonhost plants in a study involving *Vigna sinensis* (cowpea), *Phaseolus vulgaris* (French bean), *Phaseolus lunatus* (Lima bean), *Pisum sativum* (garden pea), *Vicia faba* (broad bean), *Brassica oleracea* (cabbage), *Helianthus annus* (sunflower), and *Zea mays* (maize) with three rust pathogens, *Uromyces phaseoli* var. *vignae*, *Puccinia helianthi* (sunflower rust) and *Puccinia sorghi* (Heath, 1977). In most interactions the rust pathogen frequently germinated to identify a stoma and produce an appressorium, with the majority of infections terminating following formation of a haustorial mother cell within the apoplast. In most interactions haustoria were not observed and in those rare cases were they did occur they were usually associated with plant cell death. A distribution of infection site outcomes was observed on single leaves that ranged from germinated spores unable to locate a stoma to those that produced haustoria (Heath, 1977).

### NONHOST RESISTANCE TO THE ASIAN SOYBEAN RUST PATHOGEN

As described above, *Phakopsora pachyrhizi* urediniospores germinate to produce appressoria that directly penetrate the plant epidermis. *Phakopsora pachyrhizi* urediniospores also germinate on the leaf surface of nonhost plants such as *Arabidopsis thaliana* and barley and produce appressoria (Loehrer et al., 2008; Hoefle et al., 2009). Penetration is often unsuccessful with the underlying epidermal cells producing callose enriched cell wall appositions at attempted infection sites in both species. However, numerous infection hyphae did successfully penetrate the epidermis of both species and reached underlying mesophyll cells, but the fungus did not successfully invade the mesophyll of either species (Loehrer et al., 2008; Hoefle et al., 2009). Similar to mildew pathogens these observations imply dual defense mechanisms, including both pre-invasive and post-invasive, that act against this soybean rust pathogen in nonhost interactions.

Barley plants with mutations in the *Ror1* gene, a homolog of the *Arabidopsis thaliana PEN1* gene, have much higher rates of epidermal penetration by the nonadapted soybean rust pathogen, consistent with *PEN*-mediated pre-invasive defenses also being effective in this NHR response (Hoefle et al., 2009). Similarly, infection of *Arabidopsis thaliana* plants defective in any of three *PEN* genes enabled the fungus to grow within the mesophyll intercellular spaces and occasionally produce haustoria, with this effect being most pronounced in *pen3* mutants (Loehrer et al., 2008; Langenbach et al., 2013). Pathogen growth was further enhanced in *pen* plants that were also deficient in either SA (*pen3*/*sid2; pen2/pad4/sag101*) or JA (*pen3*/*jar1*) signaling pathways, with an increase in haustoria production (Loehrer et al., 2008; Langenbach et al., 2013). An additional gene, *BRT1*, involved in phenylpropanoid metabolism was also shown to contribute to post-invasive defense in a *pen2* mutant background, again demonstrating inducible defense mechanisms (Langenbach et al., 2013).

### NONHOST INTERACTIONS OF RICE

Five cereal rust species (*Puccinia graminis* f. sp. *tritici*, *Puccinia hordei*, *Puccinia triticina*, *Puccinia striiformis* f. sp. *tritici*, and *Puccinia sorghi*) were shown to infect rice and produce all the infection structures necessary for colonization, including haustoria (**Figures 2F-H**). In some instances infection sites were large and encompassed hundreds of mesophyll cells (**Figure 2E**), however, sporulation was never observed (Ayliffe et al., 2011). Nonhost rice plants responded with active defense responses (callose deposition, reactive oxygen species production and rarely cell death) that likely play a role in limiting colonization and consequently prevent sporulation (Ayliffe et al., 2011). However, it appeared that cereal rusts were able to take up nutrients from rice to develop the relatively large infection sites observed in some cases, as the limited energy stored in rust spores is unlikely to support the observed degree of growth. Interestingly, for *Melampsora lini*, the flax rust pathogen, only 37% of spores that germinated on a rice leaf developed appressoria (compared to 92% on the host). These were often morphologically deformed or not positioned at stomata (**Figure 1A**), the normal route of penetration. The authors conclude that NHR has a component determined by basic compatibility, reflected by the phylogenetic distance between the host and the nonhost. A subsequent study (Yang et al., 2014) looked for differences in nonhost interactions between *Puccinia striiformis* f. sp. *tritici* and the two rice subspecies *japonica* (11 varieties) and *indica* (12 varieties). Germination rates were similar for all varieties, but most of the germ tubes did not recognize stomata on the *japonica* varieties, whereas successful penetration and substomatal vesicle formation was more common in the *indica* varieties. This coincided with reactive oxygen species release and hypersensitive response at attempted sites of infection in *japonica* varieties, which were not observed in *indica* varieties.

### NONHOST RUST INTERACTIONS OF *Brachypodium* spp.

*Brachypodium distachyon* is a model grass for temperate cereals such as wheat and barley due to its small genome, small stature, rapid life cycle, and relatively recent divergence from the Triticeae 35–40 million years ago (Draper et al., 2001; Bossolini et al., 2007). As *Brachypodium distachyon* (*sensu lato*) has recently been divided into the three species *Brachypodium distachyon, Brachypodium stacei*, and *Brachypodium hybridum* (López-Alvarez et al., 2012), many studies include representatives from more than one of these species. *Brachypodium distachyon* is host to a rust pathogen, *Puccinia brachypodii*, and genetic studies indicate that resistance to this pathogen is polygenically inherited (Barbieri et al., 2011, 2012). Infection of *Brachypodium* species with nonadapted rust species and ff. spp. results in macroscopic symptoms including immunity, small necrotic lesions, or the production of small sporulating pustules, depending on the rust species, *Brachypodium* species, or accession and growth conditions (Draper et al., 2001; Barbieri et al., 2012; Ayliffe et al., 2013; Figueroa et al., 2013). Lesions and pustule development have been observed on some *Brachypodium* spp. upon infection with the *Puccinia graminis* ff. spp. *tritici*, *lolii, phlei-pratensis, aveneae,* and *phalaridi* and the *Puccinia striiformis* ff. spp. *tritici, hordei,* and *bromi*, but less so with *Puccinia triticina*. Interestingly, the majority of stem rusts of the Aveneae/Poeae (*Puccinia graminis* ff. spp. *lolii*, *phlei-pratensis*, *aveneae,* and *phalaridi*) produced small sporulating pustules on most *Brachypodium* spp. accessions tested, arguing these pathosystems are closer to an intermediate host response than NHR (Ayliffe et al., 2013; Figueroa et al., 2013). In contrast, the majority of *Brachypodium* spp. accessions were immune to rust pathogens of the Triticeae (*Puccinia graminis* f. sp. *tritici* and *Puccinia striiformis* f. sp. *tritici)*, which is consistent with a true nonhost relationship. The increased susceptibility of *Brachypodium* spp. to rust pathogens of the Poeae is not consistent with the proposed equidistant phylogenetic relationship between the Brachypodieae, Poeae, and Triticeae (Soreng et al., 2007).

Microscopic analyses elucidated differences in the resistance response of *Brachypodium distachyon* to *Puccinia graminis* rusts. In one study the response was prehaustorial (Figueroa et al., 2013), whereas in a second study a range of infection site sizes was observed on single leaves. These varied from substomatal vesicles to larger sites that encompassed many mesophyll cells with frequent haustoria production and occasional sporulation (Ayliffe et al., 2013). Presumably this difference is due to a combination of the different environmental conditions, different rust isolates, and different *Brachypodium* genotypes used in each study. Frequent callose deposition and H_2_O_2_ production around infection sites was associated with resistance in the latter study. Cell death was relatively rare in most cases suggesting that hypersensitive cell death is not a major component of this defense response. Additionally, Ayliffe et al. (2013) reported similar NHR responses to *Puccinia striiformis* f. sp. *tritici* (**Figures 2C,D,I**) and that immunity segregated as a single dominant gene in one *Brachypodium distachyon* mapping family (BdTR10h × TEK4) and potentially as two dominant, linked resistance genes in a second family (BdTR13k × Bd21).

### BARLEY AND NONADAPTED RUSTS

In some cases, a vast majority of a given plant species may be immune to a pathogen species apart from a few isolated lines. This so called “near NHR” has been demonstrated in barley (*Hordeum vulgare*) with *Puccinia triticina*, *Puccinia hordei-murini*, *Puccinia hordei-secalini*, and *Puccinia persistens* (Neu et al., 2003; Jafary et al., 2008; Niks, 2014). Based on a screen of 56 *Puccinia triticina* isolates on the barley accession Bowman, Neu et al. (2003) identified a highly virulent isolate that was subsequently tested on a panel of 18 barley lines. Phenotypes ranged from immunity, to intermediate resistance (small uredinia), and one line was very susceptible (i.e., producing many pustules), demonstrating the intermediate position of this interaction on the scale from full compatibility to complete incompatibility. Even in the immune accession Cebada Capa 20% of infection units produced large infection sites and in some cases even initiated uredinia formation. Interestingly, expression of *HvNR-F6*, an ortholog of the rice PAMP receptor *Xa21* (Song et al., 1995), was upregulated after infection with the nonadapted pathogen *Puccinia triticina* and the adapted pathogen *Puccinia hordei*. The authors discuss “a continuum of resistance paradigms,” which may involve similar mechanisms, especially in cases where the pathogens or host and nonhost plants are closely related, as demonstrated in the case studied.

Numerous QTLs were found to confer resistance to *Puccinia triticina*, *Puccinia hordei-murini*, *Puccinia hordei-secalini*, and *Puccinia persistens* when inoculated on three barley doubled haploid populations (Cebada Capa × SusPtrit, Vada × SusPtrit, and Oregon Wolfe Barley Dominant × Recessive). The authors mirror this observation with the diversity of QTLs governing resistance in host systems (Jafary et al., 2008) and compare their results with the location of the major hypersensitive response gene (*Rph7*) and several partial resistance (*Rphq*) genes to the host pathogen *Puccinia hordei*. As in previous studies (Neu et al., 2003), *Rph7* was not found to be involved in resistance against these nonadapted rusts, but the *Rphq* QTLs colocalized with the QTLs identified against these pathogens. The authors conclude that qualitative *R* genes are not commonly involved in this “near NHR,” but genes conferring partial resistance to adapted pathogens may play a role in NHR (Jafary et al., 2008; Niks, 2014).

Similar polygenic interactions were observed between *Puccinia graminis* f. sp. *avenea* and barley, where transgressive segregants that allowed pathogen sporulation, albeit associated with a mesothetic response, were observed in a mapping family derived from two immune parents (Dracatos et al., 2014). In both parents clear prehaustorial resistance was identified, which involved infection hyphae touching the plant cell surface, but not penetrating the plant cell wall. Host cells responded with localized deposition of autofluorescent material at contact sites, however, no cell death was apparent (Dracatos et al., 2014). The barley prehaustorial resistance to the oat stem rust pathogen argues that either effector production and recognition is not limited to the plant–haustoria interface, such as the case of *Rpg1* in the barley-*Puccinia graminis* f. sp. *tritici* interaction (Nirmala et al., 2010, 2011), or alternatively this is an extreme PTI response. This contrasts the ability of *Puccinia graminis* f. sp. *avenea* to frequently sporulate on *Brachypodium* spp. accessions and *Puccinia graminis* f. sp. *tritici* to infect rice cells to produce large infections sites that encompass numerous mesophyll cells and contain haustoria.

## THE CONTINUUM OF INFECTION OUTCOMES

These examples demonstrate the continuum of rust infection outcomes in what is collectively categorized as NHR and range from:

(1) A basic incompatibility where the pathogen is physically incapable of efficiently infecting the host (e.g., flax rust on rice; **Figures 1A** and **2A**).
(2) Leaf entry but the rust is unable, or rarely able, to form haustoria (cereal rusts on *Arabidopsis thaliana* and bean, bean rust on wheat etc.;**Figures 1B** and **2B**). This is a very typical outcome for many nonadapted rust pathogens.
(3) Infection where all the fungal structures necessary for parasitism are produced, but sporulation never occurs (cereal rusts on rice; **Figures 1C-E** and **2D-H**).
(4) The formation of occasional tiny sporulating pustules (rusts with Triticeae hosts on *Brachypodium* spp.; **Figures 1F** and **2I**).
(5) The frequent formation of very small pustules (rusts with Aveneae/Poeae hosts on *Brachypodium* spp.).
(6) “Near NHR” (wheat leaf rust on barley).

The above observations are generalizations in that rare infection sites do develop further in each case. These observations are largely consistent with the proposed layered model of NHR mechanisms (Thordal-Christensen, 2003). It is also apparent that within each nonadapted rust pathosystem a range of infection outcomes can occur on a single leaf with a proportion of infections sites not entering the apoplast, a proportion entering but not forming haustoria, a proportion forming haustoria, and so on. Each NHR interaction is therefore a distribution of outcomes with the median outcome lying between extreme resistance and partial susceptibility depending upon the NHR pathosystem.

Unsurprisingly, in the above examples it is generally observed that the more related the host and nonhost plant species, the greater the rust pathogen colonization on the nonhost. In the case of wheat rusts, for example, the extent of infection on *Arabidopsis thaliana* was very limited, but increased on rice and even more so on *Brachypodium* spp. Presumably, elements of basic incompatibility and inappropriate plant signals decrease as host and nonhost relatedness increase. In addition, the efficacy of pathogen virulence molecules, like effectors, may also increase with this increasing relatedness, as the plant molecules targeted by these effectors either increase in similarity or a greater proportion become identical between the host and nonhost (Dong et al., 2014).

The increasing colonization of rusts on nonhosts that are more related to the pathogens’ host species seems to hold up to a point, but then terminates somewhat spectacularly with minimal pathogen growth accompanied by a hypersensitive cell death response occurring in cells containing haustoria, e.g., wheat and *Puccinia coronata* f. sp. *avenea* (Moerschbacher et al., 1990). These observations generally fit the molecular evolutionary model proposed by Schulze-Lefert and Panstruga (2011). According to this model, PTI immunity plays a key role in NHR when pathogens attempt to infect more distantly related nonhost species, but a point is reached when host and nonhost plants are each infected by similar pathogen species (Schulze-Lefert and Panstruga, 2011). So similar in fact, that these two pathogen species share common effector molecules. Hence, the nonhost plant may contain pre-existing R proteins capable of directly recognizing effectors or effector activities deployed by the nonadapted pathogen.

**FIGURE 3:**
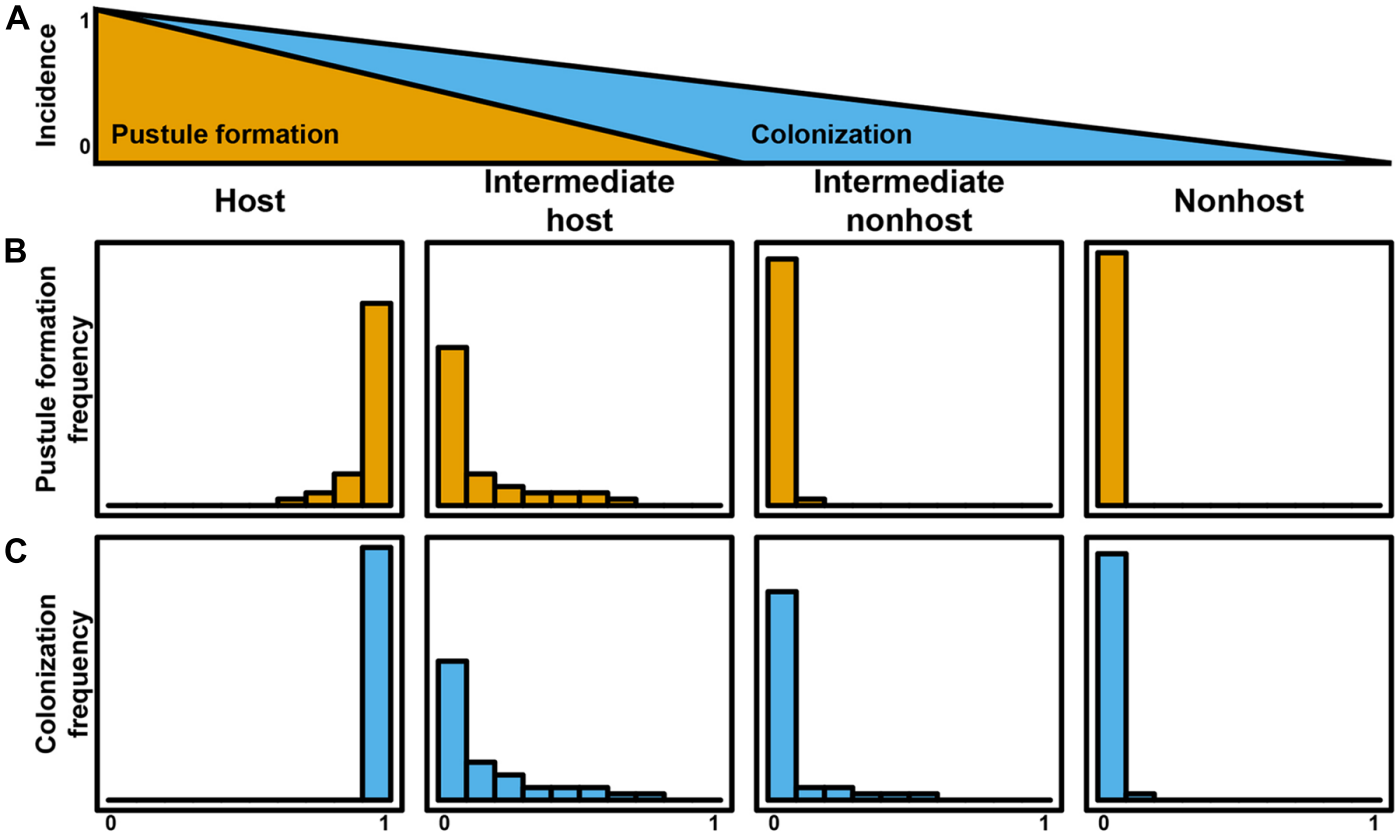
**Quantitative plant–rust interaction model for the transition from host to nonhost.** The incidence of life cycle completion of a nonadapted pathogen decreases faster than incidences of colonization **(A)**. Pustule formation frequency **(B)** and colonization frequency **(C)** define host systems, intermediate host systems, intermediate nonhost systems, and nonhost systems.

As illustrated by the different plant–pathogen interactions discussed, it is important to consider the status of an interaction based on initial differentiation of the rust, colonization, and life cycle completion. The continuum of outcomes listed above and visualized in **Figures 1** and **2** highlight that a pathogen’s ability to complete its life cycle on plant species will decrease faster than its ability to penetrate the plant and produce infection structures (**Figure 3A**). In an extreme host interaction, a panel of accessions would be severely colonized and forming pustules. Conversely, a panel of accessions from a true nonhost species would show no signs of colonization or life cycle completion by a nonadapted pathogen. We propose the terms “intermediate host” and “intermediate nonhost” to classify the continuum of rust infection outcomes observed in the transition from host to nonhost. The pathogens’ inability to penetrate the leaf or form infection structures such as haustoria (**Figures 1A,B** and **2A,B**) would be requirements for describing a plant as nonhost to a pathogen. In an intermediate nonhost, the pathogen can overcome the plants initial layers of defense and produce infection structures typically observed on hosts, but is unable to complete its life cycle (**Figures 1C-E** and **2D-H**) or sporulation is a very rare exception (**Figure 1F**). Frequent formation of small pustules (**Figures 1F** and **2I**) or the “near NHR” of wheat leaf rust on barley constitutes an intermediate host system. **Figure 3** illustrates the expected frequencies for pustule formation and colonization based on a representative panel of the genetic diversity of plant and pathogens in host, intermediate host, intermediate nonhost, and nonhost systems, respectively.

## BUT IT’S MORE COMPLICATED THAN THAT – OF COURSE

The above examples reasonably fit current evolutionary models regarding NHR. However, this is not always the case, particularly as host and nonhost species become more closely related. Histological studies of six species of the Poaceae (*Lolium perenne*, *Avena sativa*, *Hordeum vulgare*, *Triticum aestivum*, *Triticosecale,* and *Secale cereale*) inoculated with *Puccinia coronata* f. sp. *lolii*, *Puccinia coronata* f. sp. *avenea*, *Puccinia hordei*, *Puccinia triticina*, and *Puccinia melanocephala* (sugarcane rust) were undertaken and in all interactions fungi developed haustorial mother cells (Luke et al., 1987). Haustoria were never formed in any nonhost interaction with *Puccinia melanocephala*, consistent with the sugarcane host of this pathogen being the most divergent compared with the other plant species. However, *Puccinia hordei* also never formed haustoria, even though its host, barley, is relatively closely related to the other plant hosts of these rust species (Niks, 1983; Luke et al., 1987). In addition, *Puccinia melanocephala* showed poor appressorium development on barley and ryegrass (*Lolium*), but frequent appressorium development on oats, approximately equivalent to that observed on the host.

Moreover, in this study *Puccinia coronata* f. sp. *lolii* and *Puccinia triticina* produced haustoria in all nonhost species, but *Puccinia coronata* f. sp. *avenea* frequently produced haustoria on barley and rye but not wheat (Luke et al., 1987). Different infection sites on the same leaf led to variable outcomes ranging from no observable effect to hypersensitive cell death. In some cases cell death (collapsed, fluorescent cells) was not apparent in haustoria infected nonhost cells, which is not consistent with ETI induced by haustoria derived effectors providing resistance. Alternatively, it is possible that resistance is determined by recognition of secreted apoplastic effectors or conserved PAMPs. These data indicate that infection outcomes of nonadapted rust pathogens are often not entirely predictable and do not always readily fit current molecular models of NHR. A caveat is that microscopic phenotypes do not necessarily provide information on the underlying resistance mechanism. For example, the inability to produce haustoria in one pathosystem could be due to a phytoalexin while in a second pathosystem it may be due to PTI. Hypersensitive cell death based resistance is not always a hallmark of NBS–LRR mediated rust resistance (e.g., *Sr33*) and some rust resistance genes can elicit both cell death and non-cell death responses on the same infected leaf (e.g., *Sr45*; Periyannan et al., 2013). PTI may also result in cell death in some cases, for example by necrosis inducing elicitors from *Phytophthora* pathogens (Khatib et al., 2004; Larroque et al., 2013). Therefore, phenotypic similarities do not always necessarily confirm common resistance mechanisms.

## APPLICATION AND TRANSLATION OF NONHOST RESISTANCE

As described above, an often-cited advantage of NHR is that it is considered durable and broad spectrum (Thordal-Christensen, 2003; Mysore and Ryu, 2004; Schulze-Lefert and Panstruga, 2011). These two qualities of disease resistance are strongly sought after in agricultural crop plants. The possibility of transferring NHR to an agricultural host plant is therefore an attractive proposition. There are a number of examples of successful transfer of resistance from a nonhost species to a host (Wulff et al., 2011). The first example involved the transfer of the maize NBS–LRR encoding gene *Rxo1* to rice (Zhao et al., 2005). This gene was identified by the detection of maize lines that showed a strong hypersensitive response when infiltrated with the rice bacterial pathogen *Xanthomonas oryzae* pv. *oryzicola* (Zhao et al., 2004b) or by expression of a single type III effector protein from this bacterium in maize (Zhao et al., 2004a). *Xanthomonas oryzae* pv. *oryzicola* is the causal agent of bacterial leaf streak on rice, but is a non-pathogen of maize. A single maize gene that conferred the strong hypersensitive response phenotype induced in some lines was isolated by positional cloning and the NBS–LRR protein encoded by this gene provided resistance to *Xanthomonas oryzae* pv. *oryzicola* when transferred to rice. Intriguingly, *Rxo1* also provides protection in maize against an adapted maize bacterial pathogen, *Burkholderia andropogonis* (Zhao et al., 2005).

In the Brassicaceae species *Arabidopsis thaliana*, a leucine rich repeat receptor-kinase, *EFR*, recognizes a conserved, abundant bacterial translation initiation factor protein, *Ef-Tu*, to activate PTI (Zipfel et al., 2006). No equivalent receptor is present in solanaceous plants and transfer of this gene to tomato and *Nicotiana benthamiana* provided increased resistance against bacterial pathogens (*Agrobacterium tumefaciens, Pseudomonas syringae, Ralstonia solanacearum, Xanthomonas perforans*), some of which are nonadapted pathogens of *Arabidopsis thaliana* (Lacombe et al., 2010). It is of interest that a fully functional signaling pathway enabling *EFR* to function in these solanaceous species was pre-existing.

As described above, the wheat *Lr34/Yr18/Sr57/Pm38* gene encodes an ABC transporter-like plasma membrane protein that provides broad spectrum resistance to *Puccinia triticina*, *Puccinia striiformis* f. sp. *tritici*, *Puccinia graminis* f. sp. *tritici*, and *Blumeria graminis* f. sp. *tritici*. However, this resistance is both partial and only effective in adult plants (Krattinger et al., 2009). Transfer of this gene to barley provides resistance to pathogens adapted to both wheat and barley (e.g., *Puccinia graminis* f. sp. *tritici*) and pathogens adapted for colonization of barley alone (*Puccinia hordei* and *Blumeria graminis* f. sp. *hordei*; Risk et al., 2013). Associated with this resistance, however, is a necrotic response in barley seedlings and adult plants that is consistent with an accelerated senescence response making this resistance a non-viable option in barley in its current form.

These are all examples of resistance genes from one species conferring resistance in a second species against pathogens that do not parasitize the gene donor plant. Potentially, these genes may also play a role in protecting the donor plants against these nonadapted pathogen species. This conclusion, however, is entirely presumptive, as the absence of functional *Rxo1* and *Lr34* alleles in maize and wheat, respectively, do not increase their susceptibility to nonadapted pathogens (Zhao et al., 2005). Yet, redundancy in NHR mechanisms is likely to mask any individual contributions of these genes and adds difficulty in confirming their roles in NHR.

An important question is whether identifying single genes potentially involved in NHR and deploying them individually into host plants will result in durable resistance. Given the well-documented transient efficacy of these genes it seems unlikely that a single NBS–LRR encoding gene will be able to provide durable resistance, unless those members involved in NHR recognize an indispensible subset of either pathogen effector proteins or effector modifications of plant proteins. Conversely, a PRR would seem to offer a greater chance of durability given the conservation of PAMP molecules, suggesting biological constraints act upon these molecules that prevent mutation or deletion for resistance avoidance. However, at least on an evolutionary time scale, PTI pathways are ultimately suppressed by the deployment of novel effectors (Cui et al., 2009). Moreover, the durability of an APR gene like *Lr34* in providing resistance to pathogens of a different species after heterologous transfer would be very difficult to predict, as important factors such as the substrate of this transporter and the resulting mechanism of resistance are currently unknown.

An alarming possibility is that single gene deployment of NHR genes in new species may result in loss of their efficacy due to pathogen adaptation, which in turn may make the gene donor species susceptible to a previously nonadapted pathogen. Is this scenario likely? A case in point is the *Yr9* resistance gene introgressed into wheat from rye (*Secale cereale*) and widely deployed to provide resistance against *Puccinia striiformis* f. sp. *tritici*. After the introduction of *Puccinia striiformis* f. sp. *tritici* to Australia, triticale (an artificial hybrid of wheat and rye) retained its resistance to *Puccinia striiformis* f. sp. *tritici* for almost a quarter of a century until a race virulent for *Yr9* emerged. Due to the breakdown of two additional resistance genes (*YrJ* and *Yr27*), *Puccinia striiformis* f. sp. *tritici* is now affecting the triticale industry in Australia (Wellings, 2012). The interaction between *Puccinia striiformis* f. sp. *tritici* and triticale illustrates the influence that humans can have on pathogen evolution. Introgression of resistance into a major crop grown over a large area places a strong selective pressure on the pathogen and one could imagine that if a sufficient number of rye resistance genes get defeated in wheat or triticale, this could even lead to greater susceptibility of rye to *Puccinia striiformis* f. sp. *tritici*.

While in some instances very few genes confer apparent immunity to a pathogen species, in other instances it appears that NHR is polygenic. The loss of a single NHR gene in this latter case is unlikely to result in susceptibility of the donor gene species. Redundancy in NHR mechanisms to mildew pathogens has been well-established in *Arabidopsis thaliana* with penetration resistance mechanisms, contributed to by the *PEN* proteins, preceding a second layer of resistance dependent upon SA signaling, as described above (Fan and Doerner, 2012). Other forms of polygenic NHR do not involve multiple independent layers of resistance, but rather multiple loci, each conferring minor additive affects, as observed in the interaction between barley and *Puccinia triticina* (Neu et al., 2003; Jafary et al., 2008; Niks, 2014). This latter resistance, however, is unlikely to be of practical use due to the genetic complexity required to achieve useful levels of resistance.

The significance of the potential erosion of NHR is fundamentally linked to the agronomic status of the gene donor species. For example, the potential erosion of NHR by the deployment of genes from *Brachypodium* spp. to wheat seems of minimal concern given this species is of no practical agricultural significance. This process would be akin to the already well-established deployment of host resistance genes from wild grasses such as *Aegilops tauschii* and *Triticum monococcum* into wheat (McIntosh et al., 1995), with little concern for the probable future breakdown of host resistance in the wild wheat relatives. In contrast, the potential erosion of rice NHR to rust is potentially of far more significance, although the biological likelihood of such an event is entirely unknown, particularly given the likely polygenic nature of this resistance (Ayliffe et al., 2011). An obvious approach to alleviate some of these concerns would be to treat cloned NHR genes like any other *R* gene and avoid their single gene deployment.

## SUMMARY

Rust evolution has involved numerous host species jumps that involve plants with wide taxonomic divides and which in some cases have been essential for the formation of heteroecious pathogen life cycles. These host jumps demonstrate that plant NHR mechanisms to rusts can be overcome by the pathogen – but generally over long evolutionary periods of time. Modern ff. spp. offer an insight into the process of coevolution and speciation of host and nonhost plant species with rust pathogens. Phenotypically, the NHR response to rusts covers a range of interaction outcomes from basic incompatibility to active defense responses, presumably based upon the same surveillance mechanisms well-established in other pathosystems. However, phenotypic outcomes of rust infections by nonadapted pathogens can be difficult to predict and do not necessarily follow phylogenetic relationships of host and nonhost species. These phenotypic observations have been known for some time, yet, progress in understanding the molecular mechanisms of NHR in a number of plant–rust pathosystems has been made. These interactions tend toward partial resistance rather than true NHR immunity due to the more quantifiable and differential infection outcomes observed. Nonetheless, the genes underlying this resistance will be of great interest, as will be the determination of their potential application in agriculture.

## Conflict of Interest Statement

The authors declare that the research was conducted in the absence of any commercial or financial relationships that could be construed as a potential conflict of interest.
